# “Hold the retractor, that’s it?” – A retrospective longitudinal evaluation-study of the surgical and the elective tertial in the practical year

**DOI:** 10.3205/zma001727

**Published:** 2025-02-17

**Authors:** Anna Junga, Dennis Görlich, Sönke Scherzer, Meike Schwarz, Henriette Schulze, Bernhard Marschall, Jan Carl Becker

**Affiliations:** 1 University of Münster, Medical Faculty, Institute for Education and Student Affairs, Münster, Germany; 2 University of Münster, Medical Faculty, Institute of Biostatistics and Clinical Research, Münster, Germany; 3 Dortmund Fertility Centre, Dortmund, Germany

**Keywords:** medical education, surgery, practical year

## Abstract

**Introduction:**

In the context of the shortage of physicians, the practical year is an important component in the acquisition of future medical talent. Previous studies suggest that PJ students rate several satisfaction parameters significantly lower in the surgical term than in other terms. Poor perceptions of surgical topics may lead to a health care problem. The aim of the current study was to analyse in detail the specific comparison between compulsory surgical and other elective surgical terms.

**Methods:**

7762 anonymous online PJ evaluations at the Medical Faculty of Münster from 2007-2020 (RR 60.6%) were retrospectively analysed. The elective subjects were divided into operative and conservative subjects. In particular, evaluations and subjective learning gains were compared.

**Results:**

On the one hand, this study confirmed that the mandatory subject surgery was rated significantly worse than the other tertials (M_surg_=69,3, M_Int_=76,7, M_elec_=84,6; p<0,001). Among the elective subjects, the conservative subjects were also preferred and rated better than the operative subjects (30,7% vs. 69,3%; M_op_=85,9, M_kons_=81,8; p<0,001). A final comparison of the elective operative subjects and the compulsory surgical terms showed that the elective operative subjects were also rated sig. better than the compulsory surgical term (M_surg_=69,3, M_op_=85,9; p<0,001).

**Discussion:**

The compulsory surgical specialty was found to be deficient in student autonomy and supervision, which may explain the worsening recruitment problem. In order to improve the attractiveness of surgical training, a compulsory surgical elective could be introduced, where students could learn the core competencies in smaller units. This enhancement could increase the interest of future medical professionals in surgical training.

## Zusammenfassung

**Einleitung:**

Im Rahmen des Ärztemangels ist das Praktische Jahr (PJ) ein wichtiger Baustein der Nachwuchsgewinnung. Vorangegangene Studien deuten darauf hin, dass Studierende im PJ verschiedene Zufriedenheitsparameter im Chirurgie-Tertial deutlich schlechter bewerten als in anderen Tertialen. Eine schlechte Wahrnehmung der operativen Fächer kann zu einem wachsenden Versorgungsproblem führen. Ziel der aktuellen Studie war eine Detailanalyse des konkreten Vergleichs zwischen dem Chirurgie-Pflicht- und anderen operativen Wahltertialen.

**Methoden:**

Retrospektiv wurden 7762 anonyme online-PJ-Evaluationen an der Medizinischen Fakultät Münster von 2007-2020 (RQ 60,6%) ausgewertet. Die Wahlfächer wurden in operative sowie konservative Fächer unterteilt. Insbesondere wurden subjektive Gesamtbewertungen und Lernzuwachs verglichen.

**Ergebnisse:**

Diese Studie konnte zum einen bestätigen, dass das Pflichtfach Chirurgie sig. schlechter evaluiert wurde als die anderen Tertiale (M_Chir_=69,3, M_Inn_=76,7, M_Wahl_=84,6, je p<0,001). Auch innerhalb der Wahlfächer wurden die konservativen Fächer gegenüber den operativen Fächern präferiert und besser evaluiert (30,7% vs. 69,3%; M_op_=85,9, M_kons_=81,8; p<0,001). Eine abschließende Gegenüberstellung der operativen Wahlfächer und des Pflichtteils Chirurgie zeigte, dass auch die operativen Wahlfächer retrospektiv sig. besser beurteilt wurden, als das Pflichttertial Chirurgie (M_Chir_=69,3, M_op_=85,9; p<0,001).

**Diskussion:**

Das Pflichtfach Chirurgie zeigte in den Analysen Defizite in Betreuung und Selbstständigkeit der Studierenden mit der sich das verschärfende Nachwuchsproblem erklären lässt. Um die Ausbildung in operativen Fächern aufzuwerten, könnte ein operativer Wahl-Pflichtbereich eingeführt werden, in dem Studierende in kleineren Einheiten die zentralen Kompetenzen erlenen können. Diese Aufwertung könnte das Interesse des ärztlichen Nachwuchses an einer operativen Weiterbildungen stärken.

## Introduction

The final stage of the medical curriculum, the practical year (german: Praktisches Jahr/PJ), is the bridge for students into everyday clinical practice. The aim is to put into practice, under supervision, the diagnosis and treatment of diseases that have been taught in theory. According to the Medical Licensing Regulations (ÄApprO 2002), all graduates are expected to be able to work independently as a doctor after completing their studies (§ 1 Abs. 1 ÄApprO 2002). The subject of surgery is a four-month compulsory part of medical training, in which the essential basics of medical care are taught, which are of fundamental importance for every doctor. Among other things, students learn about operating theatre procedures, perioperative management, the basics of local anaesthesia and wound care, and how to recognize potentially dangerous cases. Due to the overarching importance of the above-mentioned teaching content for any specialist training, the law has made the subject of surgery a compulsory part of the PJ for all students (§ 3 Abs. 1 ÄApprO 2002).

Despite the importance of surgery for patient care, according to a 2018 study by the National Association of Statutory Health Insurance Physicians, only around a quarter of medical students surveyed would consider further specialist training in this field. 38.5% of respondents completely ruled out further training in general surgery, while the figure for trauma surgery was as high as 41.6% – and rising [1]. A continuation of this trend, combined with demographic change, threatens to exacerbate the healthcare problem in surgery [2], [3]. 

Previous studies and surveys of PJ students [4], [5], [6], [7] have already shown that the student evaluation of the compulsory tertial surgery is significantly worse than the evaluation of the compulsory tertial internal medicine and the elective specialization. In detailed analyses, this was mainly due to the better supervision and the opportunity to work independently in the latter subjects [4], [5]. Based on this, the question also arises as to whether there are also differences between conservative and surgical electives and, if so, how surgical electives differ from the compulsory surgical tertial.

Internal medicine is the second compulsory subject, but consists of eight specializations (e.g. cardiology, rheumatology, etc.). These can be used for rotations depending on the orientation of the clinic and the interests of the students. In surgery, the main areas of specialisation are often general/visceral and trauma surgery. Depending on university and the size of the teaching hospital a rotation in cardiothoracic or paediatric surgery can be available, for example [8], [9], [10], [11]. 

The practical year can have a decisive influence, both positive and negative, on the future choice of specialist training position [1]. 

This stage of training is therefore of great importance, not only in terms of content and specialization, but also in terms of recruitment. The literature has already shown a negative trend in interest in surgical training. In 2018, 32.5% of respondents were still considering further training in surgery after the pre-clinical phase. After the final year, this figure dropped to an alarming 18.1%. According to the German Medical Association, in 2022 only 10.1% of specialist qualifications will be completed in surgery (general and visceral surgery as well as orthopaedic/trauma surgery) [1], [12]. It is therefore likely that the final year, as a relevant link between study and starting work, has a negative effect on interest in further surgical training. The aim is to investigate how this effect can be counteracted to promote interest in further surgical training.

At the University of Münster, the PJ can currently be completed at 35 different teaching hospitals and hospital networks, some of which have several locations in North Rhine-Westphalia and Lower Saxony. Since the amendment of the Medical Licensing Regulations in 2013, all students in Germany have access to these places without further bureaucracy and without having to register at other universities (§ 3 ÄApprO 2002 in the version of 17 July 2012). According to the PJ office in Münster, the proportion of “external” students has remained constant in recent semesters and is well over 50%. At the end of the observation period of our study, it was possible to choose one of a total of 23 subjects during the practical year in the elective tertial at the University of Münster.

The aim of the following study was to use the existing questionnaire to specify the known deficits of the surgical tertial, using Münster as an example. The questionnaire presented here allows a more detailed categorization of these deficits. Furthermore, by clustering the elective subjects into surgical and conservative subjects, the differences between these groups were analysed and compared with the data collected for the compulsory surgery tertial. Finally, the findings were used to develop recommendations for action to improve the training of final year students.

## Methods

A total of 7762 PJ evaluations at the Medical Faculty of Münster in the period 2007-2020 were retrospectively analysed. The surveys were conducted online at the end of each tertial using the EVALuna evaluation tool [http://ms-med.evaluna.net/perl-bin/evaluna.pl?]. The anonymous survey included data on the surgical and internal medicine tertials, and the electives offered. 19 of the possible 23 electives (see table 1 (Tab. 1)) were included in the questionnaire used (see attachment 1 ). The remaining electives could only be selected at the site towards the end of the survey period and were not included in the questionnaire at that time. The survey included demographic data such as gender, age and semester of study, as well as structural data about the assessed tertial, such as the subject, the position of the tertial within the PJ, and the teaching hospital where the tertial took place. Participants were identified by a randomly assigned ID, which was linked to a student user account and could therefore be used for all repeat measurements. Using a 7-point Likert scale, students were asked whether they disagreed (*strongly disagree, disagree, somewhat disagree*) or agreed (*somewhat agree, agree, strongly agree*) with aspects of content such as supervision, subjective theoretical and practical learning success, and learning opportunities. Finally, an overall rating of the tertial was given on a 100-point scale (0=very poor, 100=very good). This is a retrospective analysis with anonymous data from the regular operation of the study organization. It is not possible to draw conclusions about individuals, groups of individuals, hospitals, etc., not least because of the large number of PJ tertials analysed. The methodological approach complies with the current version of the Declaration of Helsinki [13]. 

To answer this question, the electives were divided into surgical electives (gynaecology and obstetrics, cardiothoracic surgery, paediatric surgery, oral and maxillofacial surgery, otolaryngology, neurosurgery, orthopaedics, ophthalmology, urology) and conservative electives (neurology, pharmacology, paediatrics, pathology, microbiology, dermatology, anaesthesiology, palliative medicine, radiology) (see table 1 (Tab. 1)) based on information provided by the German Medical Association [14]. In order to counteract the bias caused by the respondents’ personal preference, a subgroup was also divided for the analysis according to the choice of compulsory subject into more “surgically interested” [op+] or more “conservatively interested” [cons+] students and their assessments of the surgical tertial were compared using a t-test.

In particular, mean scores for overall assessment, subjective practical and theoretical learning success (7-point Likert scale), and aspects that might explain any differences, such as supervision, working atmosphere, etc., were analysed. The detailed questions can be found in attachment 1 . 

If information on the supervising department was missing in a dataset, it was completely excluded from the analysis (n=522 out of a total of n=8284 datasets). Where individual anomalous data points were missing, the available data were included in further analyses where possible.

Hedges’ g effect sizes (g) were reported for pairwise comparisons based on the observed means and standard deviations. Generalized estimating equations (GEE, gamma distribution, log-link function, unstructured working correlation) with repeated measures were used to compare tertials. Students could participate in each tertial and thus be assessed in up to three repeated measures. A GEE model was calculated for each assessment question. Pairwise differences between tertials were assessed using Wald tests and results were presented as forest plots of the estimated marginalized means (with 95% confidence intervals) (see attachment 2 ). A 5% significance level was used for all analyses. Testing of all evaluation questions was exploratory and, in particular, no correction for the problem of multiple testing was used.

## Results

In this study, 7762 tertial evaluations were analysed over a period of 13 years, resulting in a response rate of 60.6%. Following the amendment of the German Medical Approbation Regulations (ÄApprO) with regard to so-called '“national mobility”', this analysis also includes external PJ students at faculty-affiliated teaching hospitals from April 2013. 56.6% of the tertials were evaluated by female students and 34.9% by male students. This distribution roughly corresponds to the ratio of women to men in medical school in germany [15].

The average age of the respondents in the evaluated tertials was 27 years.

**Figure 1:**
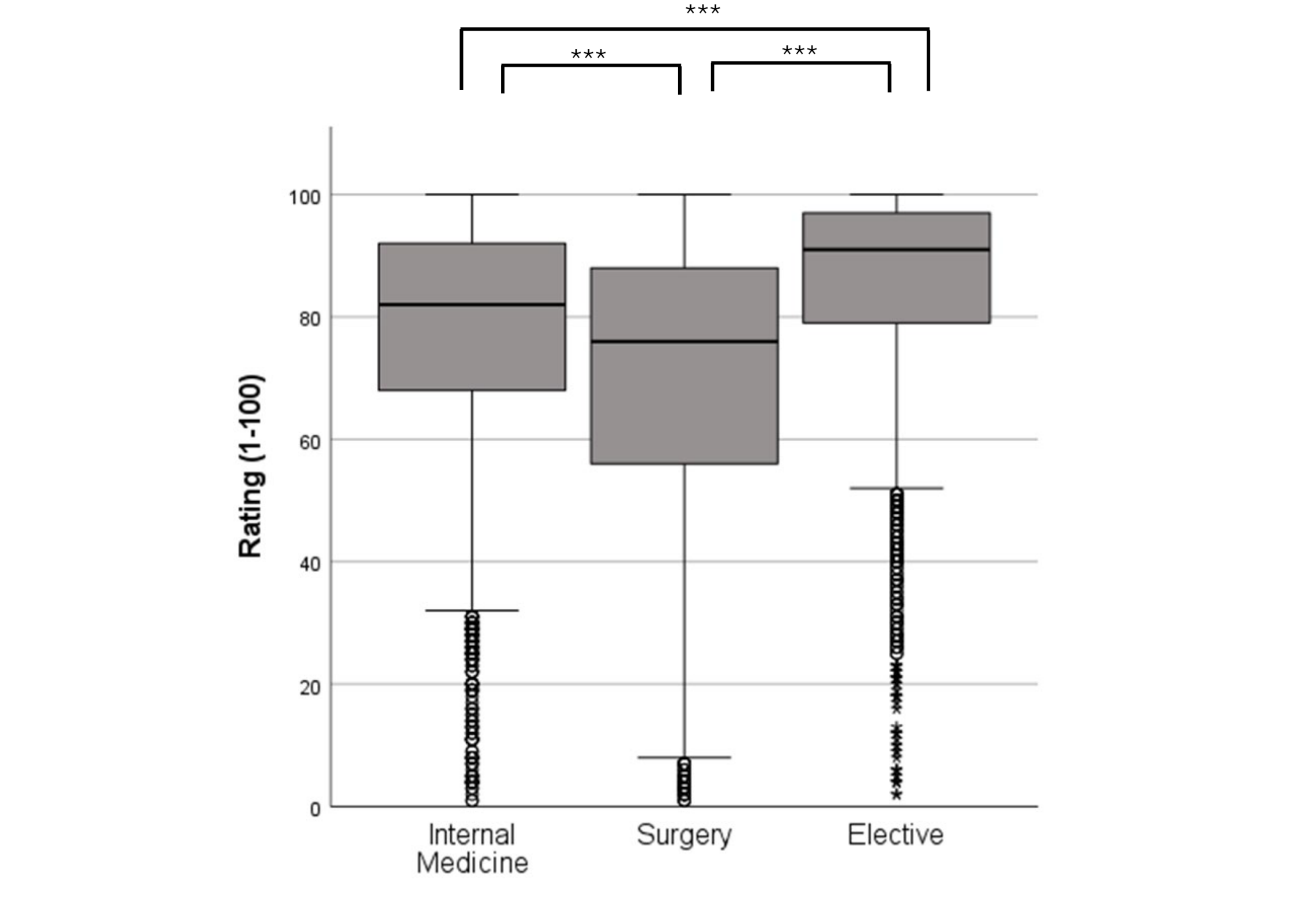
Ratings (1-100) of the different tertials; internal medicine n=2589, surgery n=2370, elective n=2679 The box represents the interquartile range, the median is marked as a line. The whiskers mark the minimum and maximum values away from the outliers, which are marked with dots (1.5 times box length) and stars (> 2.5 times box length).***=p<0,001

In this current study, it was shown that PJ students also rated the compulsory subject of surgery significantly worse in the overall assessment over the last 13 years with a mean value (M) of 69.33 points (standard deviation (SD)=23.82) than internal medicine 76.72±20.60 (M±SD; g=0.33) and the elective tertial group. The latter scored best in this comparison with a total of 84.61±17.79 (M±SD; g=0.73) (see figure 1 (Fig. 1)). Surgery was also inferior in terms of theoretical and even practical subjective learning success (4.66±1.69 for M_pracLearn_=5.26±1.66; 4.43±1.56 for M_theoLearn_=4.99±1.55). There are many comments in the free text section that support these figures: “[Surgery is] by far my worst tertial overall after two good tertials in internal medicine and neurology!”; “ACH (=general surgery), a lot of retraction holding, little teaching”.

A further breakdown of electives into conservative and operative subjects (see table 1 (Tab. 1)) shows that although the subject groups are approximately the same size (10 vs. 9 subjects), there is a clear tendency for students to choose conservative electives. Only 30.7% of students chose a surgical elective, while 69.3% chose a conservative elective. However, 38.8% of the conservative tertials were in anaesthesia alone – by far the most popular elective.

**Figure 2:**
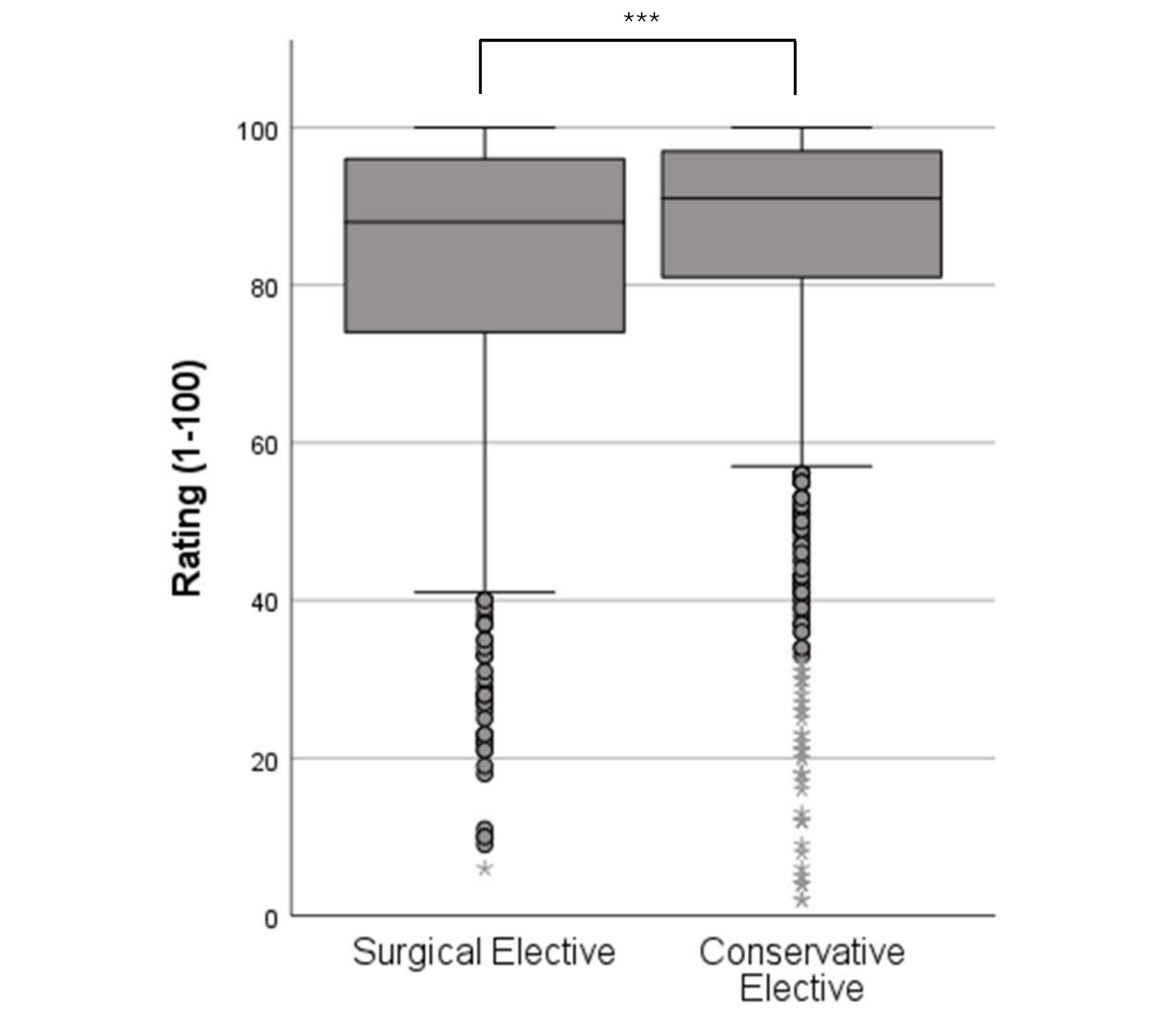
Rating (1-100) of elective terms by group; surgical electives n=794, conservative electives n=1792 (Description of the boxes in figure 1),***=p<0,001

Comparing the overall ratings of the surgical (81.82±18.97) and conservative (85.88±17.07) elective procedures, the latter were rated significantly better (M±SD; p<0.001) (see figure 2 (Fig. 2)). The effect size for this comparison is 0.22. Both subject groups separately scored significantly better than internal medicine (76.61±20.60) and even better than surgery (69.35±23.82) (M±SD; p<0.001 in each case).

There was a similar assessment of learning between the two elective groups. While the theoretical learning success of the surgical subjects was rated at 5.24±1.48, the learning success of the conservative subjects was significantly higher at 5.54±1.44 (M±SD; g=0.21; p<0.001). Practical learning success was also significantly lower in the surgical group (5.63±1.58) than in the conservative group (5.95±1.47) (M±SD; g=0.21; p<0.001).

The difference between surgery and elective surgery as a subject group was then analysed. In a head-to-head comparison, surgery scored significantly lower on the overall assessment with a score of 69.35±23.82 compared to the surgical electives with 81.86±18.97 (M±SD; p<0.001). In the area of learning success, surgery also performed significantly worse with a mean score of 4.43±1.56 vs. 5.24±1.48 for theoretical learning success and 4.66±1.70 vs. 5.63±1.60 for practical learning success (M±SD; p<0.001).

The assessment of the compulsory surgical tertial is influenced by the individual preference for surgical subjects ((op+: n=421, M=70.31 (SD=23.16) vs. cons+: n=943, M=66.90±24.76; p<0.05); g=0.14).

**Figure 3:**
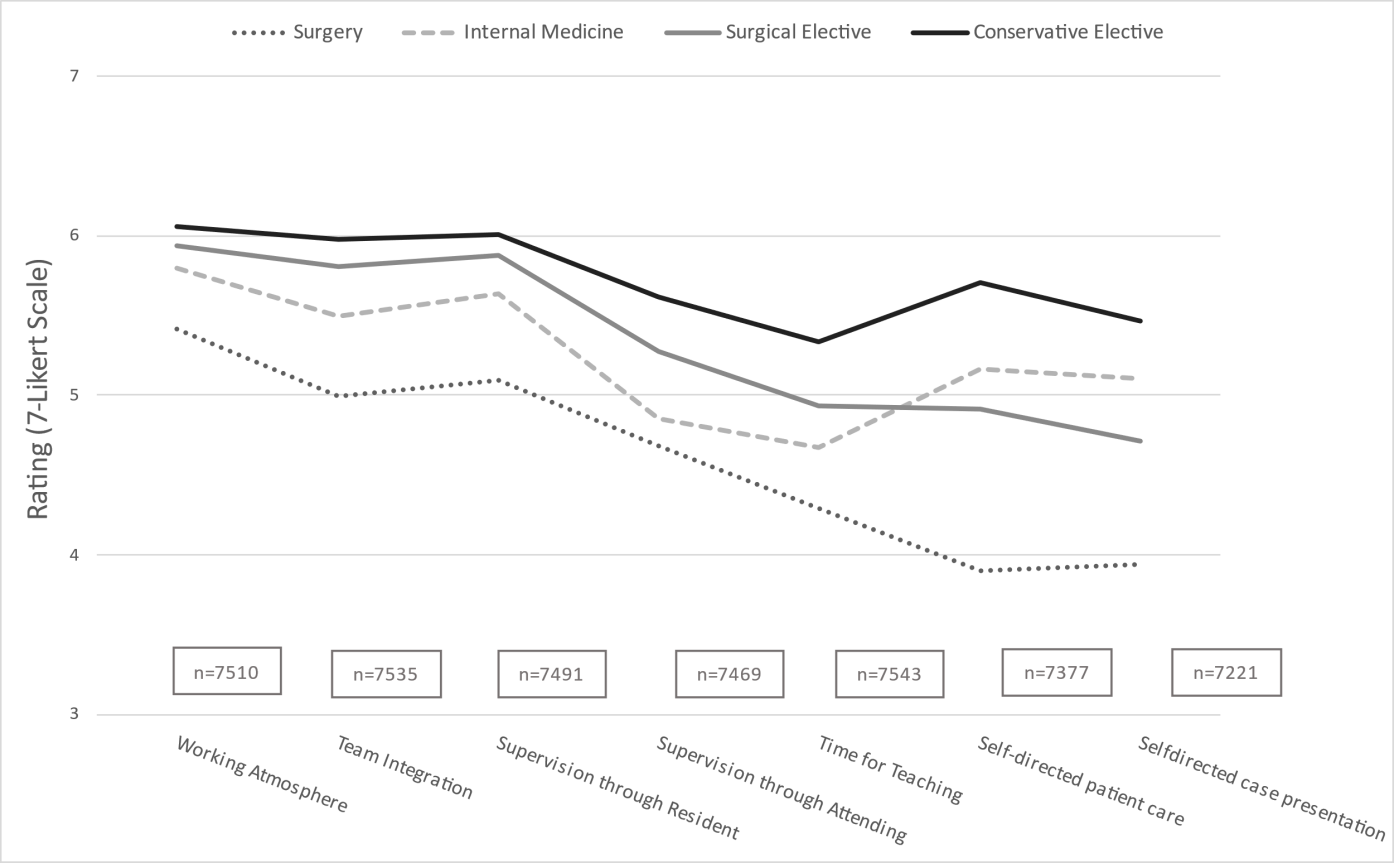
Mean values for various question items on the learning experience (Likert scale 1-7) Plotted on a scale of 3-7 for better visualisation; working atmosphere n=7510, team integration n=7535, supervision through resident n=7491, supervision through attending n=7469, time for teaching n=7543, Self-directed patient care n=7377, self-directed case presentation n=7221

The detailed analysis based on the most relevant factors according to Schwarz [16] also revealed clear differences. The differences were particularly strong in relation to the question of caring for own patients ((3.89±1.94 vs. 4.91±1.93); g=0.52) and conducting own case presentations ((3.93±1.95 vs. 4.7±1.94); g=0.40), e.g. as part of ward rounds (p<0.001 for all comparisons). A similar distribution was also found for other detailed questions (see figure 3 (Fig. 3) and attachment 2 ).

In summary, more students overall chose conservative electives than surgical electives. Compulsory surgery scored the lowest in all aspects of student evaluation. This applies to global factors such as global assessment and learning success, as well as to the detailed analyses of the learning experience. In contrast, the electives in the surgical group were rated significantly better than surgery, although worse than the electives in the conservative group.

## Discussion

This study, using a relatively large dataset of n>7000 evaluations,

1. confirmed that PJ student satisfaction and subjective learning were rated significantly lower in the compulsory surgical tertial than in the internal medicine and elective tertials, although the overall level was quite high.
2. showed that a direct comparison of conservative and surgical electives revealed a higher acceptance and better evaluation of the former, which were also chosen significantly more often.
3. showed in a subsequent comparison of the surgical elective and the compulsory surgical tertial significant differences in favour of the elective in all important factors. This forms the basis of the following discussion.

The fact that the compulsory subject of internal medicine was rated higher than surgery can be partly explained by the distribution of students’ interests. 69% of all students chose a conservative subject for their elective, as opposed to a surgical subject. Students with conservative interests also rated the compulsory surgical tertial significantly lower. The clear difference in size between the two groups reinforces this, but the overall effect can be considered weak. Another relevant point for the significantly better rating of internal medicine, with its eight specialties (often two to four internal medicine clinics per hospital), as well as the electives, could be that students in these subjects are spread across smaller functional units with the same number of beds, and personal preferences can therefore be better accommodated. In addition, electives are much more likely to meet personal preferences than compulsory subjects, which is reflected in better ratings. An expansion of the range of compulsory surgical subjects could lead to an increase in value through a potential improvement in supervision and integration. A partially expanded range of subjects can already be chosen at certain university locations, although the offer is very inhomogeneous [11]. The various campaigns and funding programs to strengthen general practice in recent years have had a positive impact [17], [18], [19]. Similar support programs could also have a positive effect on surgery. Previous studies have criticized unattractive working hours and conditions, heavy workloads and a poor working environment, among other things [1]. In this study, the poorer supervision and the lower proportion of self-directed work during the compulsory surgical tertial were also identified as disadvantages in the detailed analysis (see figure 3 (Fig. 3)). These points in particular could be greatly improved, as some funding programs have already recognized [20]. 

Consistently, the conservative elective group was rated significantly better than the surgical elective group in almost all aspects of supervision and teaching.

The differences were particularly pronounced in the introduction to self-directed work (e.g. caring for one’s own patients under supervision, presenting one’s own patients during ward rounds), which is essential for preparation for medical practice (see figure 3 (Fig. 3)). A previous study has already shown that supervision and self-directed work are particularly important for students in terms of satisfaction [5]. The current study suggests that resident supervision is generally better in conservative (elective) specialties than in surgical specialties, especially surgery. This is probably due to structural reasons, as the daily structure and staffing on the wards are more frequently interrupted by operations, and therefore supervisors may change several times a day. In addition, unlike the surgical electives, the conservative electives group also includes specialist groups from non-bedside disciplines (e.g. anaesthesia, pathology, etc.). The potentially closer supervision ratio (1:1) for structural reasons would also favour the average supervision in this specialty group.

In the final comparison of the surgical electives with the compulsory surgical term, it was found that the overall assessment and the theoretical and practical learning outcomes were also significantly worse in the compulsory surgical subject. In addition to the poorer supervision described above, possible reasons for the difference in assessment could be a higher intrinsic motivation of the students due to a conscious decision to take an elective, which may also have a positive effect on the commitment of the teachers. As described above, this effect may also play a role in the different assessment of surgery compared to internal medicine.

As part of a reform of the PJ, it would be conceivable to teach some of the surgical learning objectives prescribed in the NKLM (National Competence-Based Catalogue of Learning Objectives in Medicine) in other surgical subspecialties. These could include surgical subjects from the current elective area ('“minor surgical subjects”') in addition to the current elective subjects. Implementing this cost-neutral measure would give students more choice and better adresses their individual interests and motivations, without weakening the content. At the same time, the expanded range of subjects would lead to more students in smaller operational disciplines. This could strengthen all parts of the surgical spectrum by reducing the workload and thus improving the supervision ratio in “major” surgery. Increased satisfaction would also increase the likelihood of PJ students choosing a surgical specialty and hospital for their future careers.

This study has limitations. The analysis was not stratified by teaching hospital size. Previous analyses of the dataset showed a significant advantage for smaller hospitals in the core specialty of internal medicine and in the elective specialty, but there was no measurable effect in surgery [16], [21]. Furthermore, only students of the teaching hospitals from University of Münster were surveyed. According to the updated admission regulations, which came into effect in April 2013, the teaching hospitals of all universities are open to all PJ students nationwide. It is no longer necessary to enrol at the associated university. As a result, there is now a kind of nationwide pool of teaching hospitals, of which 35 locations (+ teaching practices and all tertiary placements abroad by Münster students) in two federal states were evaluated in this study. The proportion of students from other home universities has remained constant in recent years at well over 50%, so that a large mix of respondents and a representative sample can be assumed, at least for the densely populated area of western Germany. Furthermore, only subjective factors were surveyed in this study. Follow-up studies could be conducted in the future to analyse objective learning gains in more detail.

However, the recruitment of future residents is particularly dependent on the personal impressions and assessments of the students, so the significance of the results in this case is likely to be rather limited. In summary, student satisfaction and subjective learning outcomes were measurably worse for surgical PJ students than for all other tertials. In particular, the relevant areas of supervision and independent work performed significantly worse. An expansion of the compulsory surgical tertial in the sense of a compulsory surgical elective in the upcoming licensing regulations could lead to an improvement of the training situation and increase the attractiveness of surgical subjects.

## Funding

We acknowledge support from the Open Access Publication Fund of the University of Münster.

## Authors’ ORCIDs

- Anne Junga: [0000-0002-4165-9114]
- Dennis Görlich: [0000-0002-2574-9419]
- Sönke Scherzer: [0000-0002-7197-2101]
- Henriette Schulze: [0009-0001-4364-7141]
- Bernhard Marschall: [0000-0002-1354-8687]

## Competing interests

The authors declare that they have no competing interests. 

## Supplementary Material

Questionnaire “Evaluation of practical year”

Figure S4

## Figures and Tables

**Table 1:**
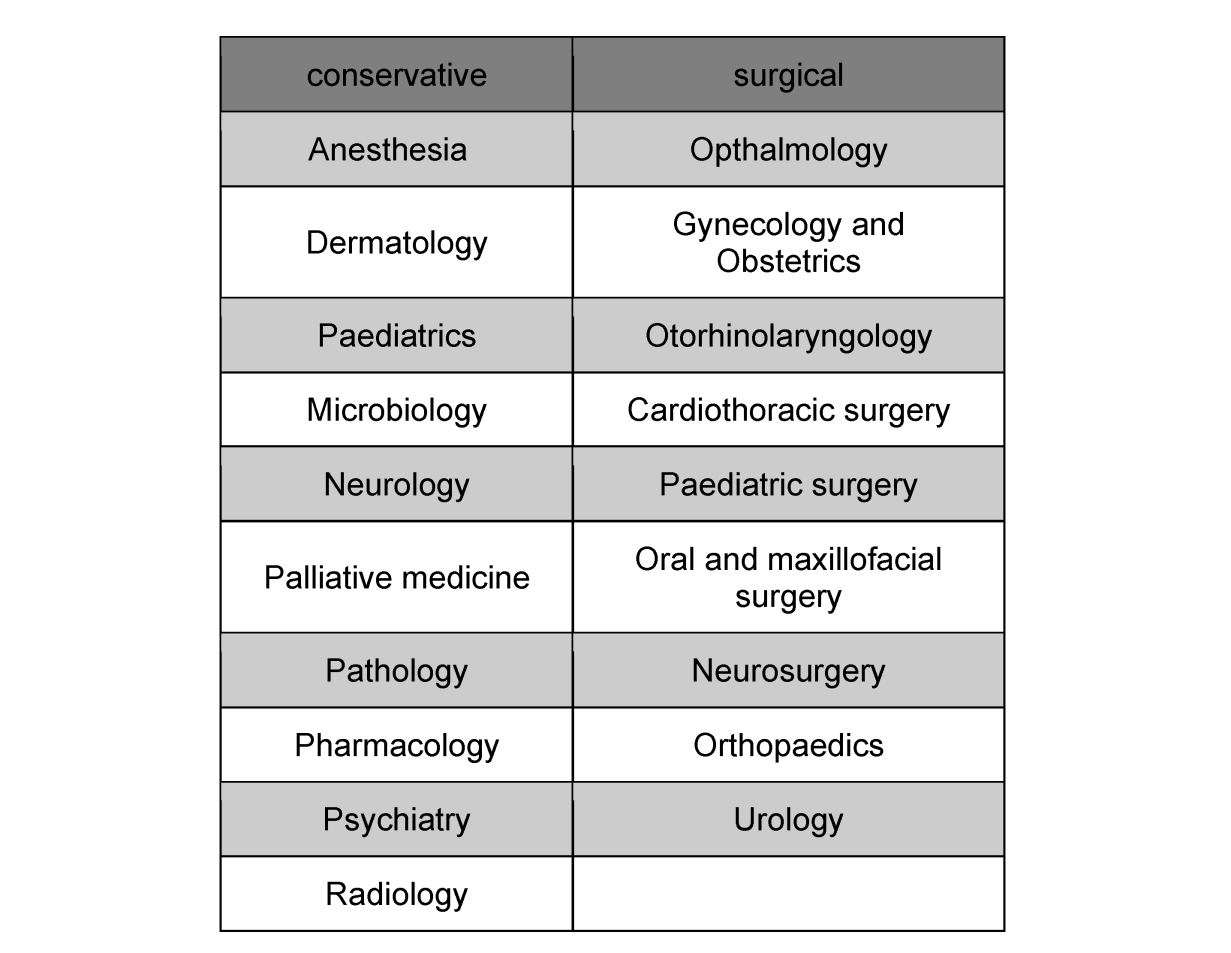
PJ electives divided into subject groups with surgical and conservative specialisations

## References

[1] Kassenärztliche Bundesvereinigung (2018). Berufsmonitoring Medizinstudierende 2018.

[2] Berufsverband Niedergelassener Chirurgen (2011). Ärztemangel und wachsender Versorgungsbedarf – wer behandelt künftig chirurgische Patienten?.

[3] Ansorg JU (2010). Nachwuchsmangel und Nachwuchsförderung in der Chirurgie.

[4] Schloßbauer A, Marschall B, Becker JC (2019). Zunahme der studentischen Zufriedenheit im PJ – eine Auswirkung der Änderungen von gesetzlichen Rahmenbedingungen?.

[5] Becker JC, Tennie M, Marschall B (2016). Zufriedenheit und Lernerfolg im Praktischen Jahr – im Wesentlichen eine Frage der Betreuung.

[6] Elsenhans I (2012). PJ-Umfrage 2014: Tolle Ausbildung oder schnöde Ausbeutung?. via medici.

[7] Rostan U (2007). PJ-Umfrage 2007: Lehrjahre sind keine Herrenjahre. via medici.

[8] Universitätsklinikum Münster Praktisches Jahr am UKM (PJ). Ausbildungsablauf.

[9] Uniklinik Köln, Klinik und Poliklinik für Allgemein-, Viszeral-, Tumor- und Transplantationschirurgie Praktisches Jahr.

[10] Universitätsklinikum Freiburg Chirurgie - Praktisches Jahr.

[11] Oppermann N, Weitz J, Reißfelder C, Mees ST (2018). Das chirurgische Tertial im praktischen Jahr – Status quo 2017. Zentralbl Chir.

[12] Bundesärztekammer (2023). Ärztestatistik zum 31. Dezember 2022. Bundesgebiet gesamt.

[13] Weltärztebund (WMA) (1964). Deklaration von Helsinki - Ethische Grundsätze für die medizinische Forschung am Menschen. Verabschiedet von der 18. WMA-Generalversammlung, Juni 1964 Helsinki (Finnland).

[14] Bundesärztekammer (2023). (Muster-)Weiterbildungsordnung 2018 in der Fassung vom 29.06.2023.

[15] Statistisches Bundesamt (2021). Studierende insgesamt und Studierende Deutsche im Studienfach Medizin (Allgemein-Medizin) nach Geschlecht. ange Reihen mit Jahresergebnisse ab 1975. Tabellen zu Bildung und Forschung mit Originalwerten und Veränderungsraten.

[16] Schwarz M (2018). Zufriedenheit und Lernerfolg der Studierenden im Praktischen Jahr an der Medizinischen Fakultät Münster - im Wesentlichen eine Frage der Betreuung.

[17] Richter-Kuhlmann E, Rieser S (2015). Allgemeinmedizin: Ein Fach im Aufwind. Dtsch Arztebl.

[18] (2015). Nachwuchsförderung in der Allgemeinmedizin erfolgreich. aerzteblatt.de.

[19] Kassenärztliche Vereinigung Westfalen-Lippe, Kassenärztliche Vereinigung Nordrhein (2023). PJ-Förderung, Richtlinien für Stipendien.

[20] Ansorg J, Hoffmann R (2019). Mastertrainer werden. Orth Unfallchir.

[21] Schloßbauer A (2021). Welchen Einfluss haben Rahmenbedingungen auf die Ausbildung Medizinstudierender im Praktischen Jahr? Eine retrospektive Analyse vor dem Hintergrund eines sich wandelnden Gesundheitssystems.

