# Supplementary material for: “Hold the retractor, that’s it?” – A retrospective longitudinal evaluation-study of the surgical and the elective tertial in the practical year
Source: GMS J Med Educ. 2025 Feb 17;42(1):Doc3. doi: 10.3205/zma001727 (PMC12086243; doi:10.3205/zma001727)
Supplement: Questionnaire “Evaluation of practical year” [file JME-42-3-s-001.pdf]

Attachment 1: Questionnaire “Evaluation of practical year”

General information
10.5.1 Which PJ cohort do you belong to?
17 PJ cohort “Franklin”
18 PJ cohort “Galenus”
19 PJ cohort “Hounsfield”
20 PJ cohort “Ipsen”
21 PJ cohort “Jessenius”
22 PJ cohort “Kocher”
23 PJ cohort “Levi-Montalcini”
24 PJ cohort “Mansfield”
25 PJ cohort “Nightingale”

10.10.1 What is the specialisation of the tertial being assessed?
tertial to be assessed?
Anaesthesiology
Ophthalmology
Surgery
Dermatology
Gynaecology and obstetrics
Otorhinolaryngology
Internal medicine
Paediatrics
Paediatric surgery
Oral and maxillofacial surgery
Neurosurgery
Neurology
Orthopaedics
Psychiatry
Radiology
Thoracic, cardiac and vascular surgery
Trauma surgery
Urology
Other

10.10.2 At which teaching hospital did you complete the tertial?
Universitätsklinik Münster
Klinikum Arnsberg
St. Franziskus-Hospital Ahlen
Ev. Krankenhaus Bielefeld gGmbH
Städtische Kliniken Bielefeld gem. GmbH - Klinikum Mitte
Christophorus Kliniken Coesfeld
Klinikum Dortmund gGmbH
St. Elisabeth Hospital Gütersloh
Klinikum Gütersloh
St. Barbara-Klinik Hamm
St. Marien-Hospital Hamm gGmbH
Klinikum Ibbenbüren
St. Bonifatius-Hospital Lingen
Dreifaltigkeits-Hospital gem. GmbH Lippstadt

St. Marien-Hospital, Lünen
Clemenshospital Misericordia gGmbH Münster
St. Franziskus-Hospital GmbH Münster
Herz-Jesu-Krankenhaus Hiltrup GmbH
Raphaelsklinik Münster
Euregio Kliniken Nordhorn
Klinikum Osnabrück
Franziskus-Hospital Harderberg
Stiftung Mathias-Spital Rheine
Katholisches Klinikum Ruhrgebiet Nord (KKRN)
Klinikum Stadt Soest
Marienhospital Steinfurt gGmbH
Katharinen Hospital Unna
Josefs Hospital Warendorf
Marien-Hospital Wesel
Teaching practice in general medicine
Germany Other faculty
abroad
Other faculties

Why did you choose this teaching hospital? (multiple answers possible)
10.20.1 I was assigned to this hospital against my choice
10.20.2 The known good teaching performance
10.20.3 The known good working atmosphere
10.20.4 I would like to work in this hospital
10.20.5 The location (e.g. close to home)
10.20.6 The recreational value of the location
10.20.7 Interest in a different healthcare system (abroad)

Please rate the following statements on a scale of 1-7
1 = completely inapplicable 2= inapplicable 3 = rather inapplicable
4 = neither
5 = rather true 6 = true 7 = completely true

Information on supervision and working atmosphere
20.10.1 I felt well integrated into routine medical and clinical operations.
20.10.2 The working atmosphere on the ward was pleasant.
20.10.3 The supervision by the senior physicians in training was very good.
20.10.4 The support provided by the training ward doctors was very good.
20.10.5 The doctors had enough time for teaching
20.10.6 The co-operation with the non-medical staff was very good.

Practical training
30.10.1 My supervising doctors gave me good guidance in learning practical skills.
30.10.2 I was able to participate regularly in ward rounds.
30.10.3 I was regularly assigned (under supervision) to care for my own patients.
30.10.4 I regularly presented my own patient cases (Department meeting, ward round)
30.10.5 I was deployed in various areas of functional diagnostics (e.g. sonography, endoscopy).
30.10.6 I regularly dictated medical or discharge letters.
30.10.7 The activities I carried out were regularly discussed.
30.10.8 I rate the learning effect of this tertial very highly in terms of my practical medical skills.

Theoretical training
30.20.1 In-clinic PJ courses were offered regularly.
30.20.2 I always had the opportunity to attend these training events.
30.20.3 The quality of the PJ courses was very good.
30.20.4 In addition, my theoretical knowledge was regularly promoted
30.20.5 I had sufficient time for self-study.
30.20.6 I rate the learning effect of this tertial very highly in terms of my theoretical skills.

Information on working conditions:
50.1.1 When did you start work (on average)?
Answers from 06:45 h - 10:00 h quarter-hourly possible
50.1.2 When did you finish work (on average)?
Answers from 13:45 h to 22:00 h quarter-hourly possible
50.1.3 How many night shifts did you work on average per month?
Answers from 0 to 15 possible
50.1.4 Was this voluntary?
yes
no

50.1.5 How many of these were on weekends (Sat./Sun.) or public holidays per month?
Answers from 0 to 15 possible

50.1.6 Was there time off in compensation?
yes
no

50.1.7 How often did you change wards during the tertial (rotation)?
Answers from 1 time to 12 times or ‘more than 40 times’ possible

Statements on working hours
Give an estimate of your proportionate working time for the following areas in per cent.
(Answers possible from 0% to 100% in 5% steps)
50.2.1 self-directed medical activities:
50.2.2. medical activities under supervision:
50.2.3. attending ward rounds:
50.2.4 non-medical activities:
50.2.5. attending OR:
50.2.6. self-study:
50.2.7. teaching events:
50.2.8. did you feel more like an employee or more like a trainee during the tertial?
trainee?
Scale: 1 (employee) - 10 (trainee)

Statements on self-study
55.1.1 How many hours per week on average did you have available for self-study?
Answers from 0 to 20 hours possible

55.1.2 How did you use the time for self-study?
By the hour
As a study day
Several days at a time

55.1.3 Where did you do your self-study?
At home
Mostly at home, partly at the teaching hospital
Mostly at the teaching hospital, partly at home
At the teaching hospital

55.1.4 Please evaluate the following statement: I was able to use the self-study time to train for the second medical exam
Scale: 1 (completely inapplicable) - 7 (completely applicable)

55.1.5 According to the current state of your considerations, when do you plan to take the second part of the medical exam?
Directly after completing the practical year
After a semester off to prepare for the examination
After two or more semesters off
I don't know yet.

Personal data
60.1.1 What gender are you?
female
male

60.1.2 How many semesters were you studying when you entered the tertial?
Answers from 10 to 25 or ‘over 25’ possible

60.1.3 How old were you when you started the tertial?
Answers from 23 to 40 or ‘over 40’ possible

Final assessment of the PJ tertial
60.2.1 In conclusion, please rate the tertial on a free scale from:
0 = very good to 100 = very bad

For the decision-making of your colleagues, please indicate your most important impressions here.
60.2.2 Additional question: Positive experiences:
60.2.3 Additional question: Negative experiences:
