# Supplementary material for: “Hold the retractor, that’s it?” – A retrospective longitudinal evaluation-study of the surgical and the elective tertial in the practical year
Source: GMS J Med Educ. 2025 Feb 17;42(1):Doc3. doi: 10.3205/zma001727 (PMC12086243; doi:10.3205/zma001727)
Supplement: Figure S4 [file JME-42-3-s-002.pdf]

Attachment 2: Figure S4

(a)

Question	Tertial	N Students	N Questionnaires	Mean(95%CI)	Comparison vs. Surgery	Comparison vs. Internal medicine	Comparison vs. Surgery elective	Comparison vs. Conservatve elective
Evaluation	Surgery	4296	2351	69.33(68.37;70.29)	0	p<0.01	p<0.01	p<0.01
Evaluation	Internal medicine	4296	2582	76.71(75.92;77.52)	7.39(6.20;8.57)	0	p<0.01	p<0.01
Evaluation	Surgery elective	4296	794	81.82(80.51;83.15)	12.49(10.88;14.11)	5.1(3.57;6.63)	0	p<0.01
Evaluation	Conservative elective	4296	1792	85.89(85.1;86.68)	16.56(15.35;17.77)	9.17(8.07;10.27)	4.07(2.53;5.61)	0

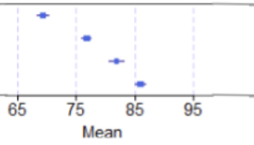

(b)

Question	Tertial	N Students	N Questionnaires	Mean(95%CI)	Comparison vs. Surgery	Comparison vs. Internal medicine	Comparison vs. Surgery elective	Comparison vs. Conservatve elective
Working atmosphere	Surgery	4301	2350	5.42 (5.36;5.48)	0	p<0.01	p<0.01	p<0.01
Working atmosphere	Internal medicine	4301	2587	5.79 (5.74;5.85)	0.38(0.31;0.45)	0	p<0.05	p<0.01
Working atmosphere	Surgery elective	4301	787	5.93 (5.84;6.03)	0.51(0.41;0.62)	0.13(0.03;0.24)	0	p<0.05
Working atmosphere	Conservative elective	4301	1786	6.06 (6.00;6.12)	0.65(0.56;0.73)	0.27(0.19;0.34)	0.13(0.02;0.25)	0
successful theoretical learning	Surgery	4299	2352	4.42 (4.36;4.49)	0	p<0.01	p<0.01	p<0.01
successful theoretical learning	Internal medicine	4299	2583	5.05 (4.99;5.11)	0.63(0.55;0.70)	0	p<0.01	p<0.01
successful theoretical learning	Surgery elective	4299	788	5.23 (5.13;5.34)	0.81(0.69;0.93)	0.18(0.07;0.30)	0	p<0.01
successful theoretical learning	Conservative elective	4299	1799	5.55 (5.48;5.62)	1.13(1.04;1.22)	0.5(0.42;0.58)	0.32(0.2;0.44)	0
successful practical learning	Surgery	4302	2349	4.65(4.58;4.72)	0	p<0.01	p<0.01	p<0.01
successful practical learning	Internal medicine	4302	2585	5.22(5.16;5.29)	0.57(0.49;0.66)	0	p<0.01	p<0.01
successful practical learning	Surgery elective	4302	794	5.63(5.52;5.74)	0.97(0.85;1.10)	0.4(0.28;0.52)	0	p<0.01
successful practical learning	Conservative elective	4302	1798	5.95(5.89;6.02)	1.3(1.21;1.39)	0.73(0.64;0.82)	0.33(0.20;0.46)	0

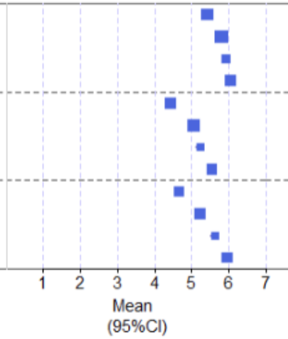

(c)

Question	Tertial	N Students	N Questionnaires	Mean(95%CI)	Comparison vs. Surgery	Comparison vs. Internal medicine	Comparison vs. Surgery elective	Comparison vs. Conservatve elective
Supervision throug hattending	Surgery	4316	2535	4.29(4.23;4.36)	0	p<0.01	p<0.01	p<0.01
Supervision throug hattending	Internal medicine	4316	2588	4.67(4.61;4.74)	0.38(0.30;0.47)	0	p<0.01	p<0.01
Supervision throug hattending	Surgery elective	4316	792	4.91(4.80;5.03)	0.62(0.49;0.76)	0.24(0.11;0.37)	0	p<0.01
Supervision throug hattending	Conservative elective	4316	1810	5.35(5.27;5.42)	1.05(0.96;1.15)	0.67(0.58;0.77)	0.43(0.29;0.57)	0
Team integration	Surgery	4316	2350	4.98(4.92;5.05)	0	p<0.01	p<0.01	p<0.01
Team integration	Internal medicine	4316	2588	5.5(5.45;5.56)	0.52(0.44;0.60)	0	p<0.01	p<0.01
Team integration	Surgery elective	4316	790	5.8(5.71;5.9)	0.82(0.71;0.93)	0.3(0.19;0.41)	0	p<0.01
Team integration	Conservative elective	4316	1807	5.98(5.92;6.04)	1(0.91;1.08)	0.48(0.40;0.56)	0.18(0.06;0.29)	0
Self-directed patient care	Surgery	4260	2325	3.89(3.81;3.97)	0	p<0.01	p<0.05	p<0.01
Self-directed patient care	Internal medicine	4260	2580	5.16(5.09;5.23)	1.27(1.18;1.37)	0	p<0.01	p<0.01
Self-directed patient care	Surgery elective	4260	782	4.91(4.78;5.04)	1.02(0.87;1.17)	-0.25(-0.4;-0.11)	0	p<0.01
Self-directed patient care	Conservative elective	4260	1690	5.72(5.64;5.8)	1.83(1.72;1.94)	0.56(0.46;0.66)	0.81(0.66;0.97)	0
Self-directed case presentation	Surgery	4219	2322	3.93(3.86;4.01)	0	p<0.01	p<0.01	p<0.01
Self-directed case presentation	Internal medicine	4219	2576	5.09(5.03;5.16)	1.16(1.06;1.26)	0	p<0.01	p<0.01
Self-directed case presentation	Surgery elective	4219	784	4.70(4.57;5.84)	0.77(0.61;0.92)	-0.39(-0.54;-0.24)	0	p<0.01
Self-directed case presentation	Conservative elective	4219	1539	5.47(5.38;5.56)	1.53(1.42;1.65)	0.37(0.27;0.48)	0.77(0.60;0.93)	0
Supervision through resident	Surgery	4301	2355	5.09(5.02;5.15)	0	p<0.01	p<0.01	p<0.01
Supervision through resident	Internal medicine	4301	2576	5.64(5.59;5.69)	0.55(0.47;0.63)	0	p<0.01	p<0.01
Supervision through resident	Surgery elective	4301	788	5.88(5.79;5.98)	0.8(0.68;0.91)	0.24(0.13;0.35)	0	p<0.05
Supervision through resident	Conservative elective	4301	1772	6.01(5.95;6.08)	0.93(0.84;1.01)	0.37(0.29;0.45)	0.13(0.02;0.25)	0

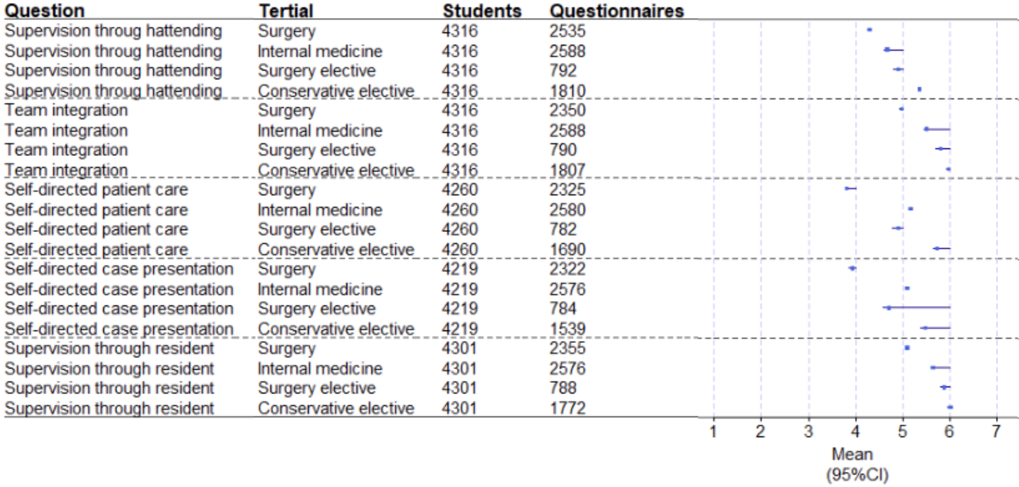

Attachment 2 to Junga A, Görlich D, Scherzer S, Schwarz M, Schulze H, Marschall B, Becker JC. *"Hold the retractor, that's it?" – A retrospective longitudinal evaluation-study of the surgery and the elective tertial in the practical year*. GMS J Med Educ. 2025;42(1):Doc3. DOI: 10.3205/zma001727

Figure S4:(a) Forest plot for the factor overall assessment for the subject groups analysed. n-numbers of the sample surveyed, mean values (1-100) with 95% CI and difference with 95% CI and significance level. (b) Forest plot of the factors ward climate, theoretical and practical learning success for the subject groups analysed. (c) Forest plot on the factors for time for training, integration into the team, care of own patients, independent case presentations and care by ward physicians for the subject groups analysed. n-numbers of the sample surveyed, mean values (1-7) with 95% CI and difference with 95% CI and significance level.
